# EEG functional connectivity in infants at elevated familial risk for autism spectrum disorder

**DOI:** 10.21203/rs.3.rs-2901872/v1

**Published:** 2023-05-15

**Authors:** Christian O’Reilly, Scott Huberty, Stefon van Noordt, James Desjardins, Nicky Wright, Julie Scorah, Sara Jane Webb, BASIS team, Mayada Elsabbagh

**Affiliations:** 1 Department of Computer Science and Engineering, University of South Carolina, Columbia, SC, USA.; 2 Artificial Intelligence Institute of South Carolina, University of South Carolina, Columbia, SC, USA.; 3 Carolina Autism and Neurodevelopment Research Center, University of South Carolina, Columbia, SC, USA.; 4 Azrieli Centre for Autism Research, Montreal Neurological Institute-Hospital, McGill University, Montreal, Canada.; 5 Department of Psychology, Mount Saint Vincent University, Halifax, Nova Scotia, Canada; 6 Compute Ontario, St. Catharines, Canada.; 7 Department of Psychology, Manchester Metropolitan University, Manchester, UK.; 8 Seattle Children’s Research Institute, Seattle, Washington, WA, USA.; 9 Birkbeck, University of London, London, UK.

**Keywords:** Autism spectrum disorder, functional connectivity, sex differences, infants, source reconstruction, electroencephalography, ADOS, sibling studies, longitudinal

## Abstract

**Background:**

Many studies have reported that autism spectrum disorder (ASD) is associated with atypical structural and functional connectivity. However, relatively little is known about the development of these differences in infancy and on how trajectories may vary between sexes.

**Methods:**

We used the International Infant EEG Platform (EEG-IP), a high-density electroencephalogram (EEG) dataset pooled from two independent infant sibling cohorts, to characterize such neurodevelopmental deviations during the first years of life. EEG was recorded at 6, 12, and 18 months of age at typical (N=97) or high familial risk for ASD (N=98), determined by the presence of an older sibling with a confirmed ASD diagnosis. We computed the functional connectivity between cortical EEG sources during video watching using the corrected imaginary part of phase-locking values.

**Results:**

Our findings showed low regional specificity for group differences in functional connectivity but revealed different sex-specific trajectories between females and males in the group of high-risk infants. Specifically, functional connectivity was negatively correlated with ADOS calibrated severity scores, particularly at 12 months for the social affect score for females and for the restrictive and repetitive behaviors for males.

**Limitations:**

This study has been limited mostly due to issues related to the relatively small effective sample size inherent in sibling studies, particularly for diagnostic group comparisons.

**Conclusions:**

These results are consistent with sex differences in ASD observed in previous research and provide further insights into the role of functional connectivity in these differences.

## Background

Heterogeneity in the causes, symptoms, and impact of autism spectrum disorder (ASD) represent a fundamental challenge to preclinical, clinical, and translational research. While genetic and non-genetic factors contribute to autism susceptibility (1,2), these factors are also likely to contribute to susceptibility for a broader range of neurodevelopmental disorders. Adding a layer of complexity, these factors interact over time, modifying early brain development and leading to heterogeneous outcomes in terms of distinct functional, cognitive, and language dimensions that are not well captured by narrow diagnostic categories (3–6).

Biological sex has emerged as an important contributor to this heterogeneity. The presence of sex-based differences in the prevalence and clinical presentation of autism is well-established (7). There is also a consensus about systematic sex differences in the timing of early developmental milestones (though the relevance of these differences remains disputed (8)). For example, girls are reported to develop language and communication abilities slightly earlier than boys (9,10). With respect to autism, the timing of early milestones is predictive of later language and cognitive outcomes (11), and the interaction between biological sex and other developmental factors may account for the delayed ASD diagnosis in girls compared to boys (12).

Biological sex factors may also play an important role in the neurobiology of autism (13), and there have been increased efforts to better understand brain development as it relates to autism and its many developmental pathways. Functional connectivity — a measure of statistical dependencies between the activity in distinct brain regions — is of particular interest because the emergence of functional networks occurs during infancy, when brain plasticity is at its peak and autism symptoms begin to emerge (14). In the general population, higher functional connectivity is observed in females relative to males for whole-brain connectivity and in functionally distinct networks, including the default mode network and the central executive network (15,16). A systematic review revealed that overall and despite the inconsistency in findings, a general pattern of long-range EEG and magnetoencephalograpy (MEG) underconnectivity distinguishes autistic from neurotypical individuals (17). These participant samples have often been heavily skewed toward males, resulting in a gap in understanding the development of functional networks in autism in males relative to females. The few studies that have explored the effect of biological sex on functional connectivity as it relates to autism have reported that autistic females show increased connectivity compared to both autistic males (18) and neurotypical females (19). Further, one study reported reduced functional connectivity associated with ASD symptoms specifically in females (16).

Once expanded and replicable in independent samples, these findings would lend support to the “female protective effect” (FPE) which states that compared to males, more severe etiological factors are necessary for autistic expression in females (20–22). FPF has been proposed to explain the relatively higher prevalence of autism in males, a discrepancy that appears to remain even after accounting for known ascertainment biases (23).

An effective way to examine the FPE is to study very early development in autism, before risk signs become compounded and amplified by atypical interactions within the brain and with the external environment. Despite the rapid increase in studies on early trajectories of brain development in infants who later develop autism (14), little is known about early functional connectivity, particularly as it relates to autism and biological sex. Only a few ASD studies have estimated connectivity in infancy (24–28). While the findings have not been conclusive, current evidence suggests that cortical network maturation differs in autistic individuals, with initial overconnectivity within the first year of life followed by underconnectivty beginning in toddlerhood (17). To date, very few infant-sibling studies have included biological sex as a variable of interest when studying functional connectivity in ASD, with no significant sex-related results (24,25). This situation might be due in part to the study of biological sex in sibling studies being complicated by the relatively low number of children who go on to develop autism and the high sex imbalance (mostly male) in the diagnosed subsample.

In the current study, we focus on the properties of the functional connectome in early development to better understand variations between infants with an elevated likelihood for autism (ELA; assessed by the presence of a sibling with ASD) compared to a control group of infants with a typical likelihood of autism (TLA; no family history of ASD), while also accounting for biological sex. To address the challenges associated with the heterogeneity of this condition, we utilize the International Infant EEG Platform (EEG-IP). EEG-IP addressed the aforementioned challenges by pooling and standardizing EEG recordings from two studies of ELA and TLA infants (Desjardins et al., 2021; van Noordt et al., 2020). Further, besides reporting results for categorical outcomes, we also perform dimensional analyses using Autism Diagnostic Observation Schedule (ADOS) severity scores to addressed previous critics regarding the use of categorical diagnostic outcomes. Previous studies have found categorical analyses to mask significant heterogeneity in the nature and severity of symptoms for children who develop autism (31). This issue also extends to children who do not receive an ASD diagnosis but experience problems in other developmental domains such as language and attention (32). Moreover, dimensional analysis allow us integrate developmental trajectories in children at risk for ASD who do or do not experience challenges and gain further insights into resilience processes (14).

## Methods

### Sample

The sample used for this study was taken from the EEG Integrated Platform (EEG-IP; van Noordt et al., 2020), which includes ELA and TLA infants. We used data from 195 participants across two sites: the Seattle Children’s Hospital (Seattle, Washington, USA) and Birkbeck University (London, UK). EEG was collected around 6, 12, and 18 months of age. Clinical diagnostic assessments included Autism Diagnostic Observation Schedule ADOS and were confirmed by clinical judgment. ADOS was administrated at around 24 months of age for both sites (ELA only) and around 36 months (ELA and TLA) for the London site. ADOS calibrated severity scores (33) were calculated using ADOS scores gathered around 24 months. Participants with an unknown outcome (n=3) or TLA later diagnosed with ASD (n=3) were excluded from the analysis. The participant demographics are summarized in Table 1.

### EEG acquisition and pre-processing

EEG was collected as infants watched videos presented on a computer monitor while seated on their caregivers’ lap in a dark room. Infants from the Seattle study viewed a series of age-appropriate videos that included brightly colored toys that moved and produced sounds, alternated with an adult female singing nursery rhymes. Each of these video sets was approximately 60 seconds in duration. Infants from the London sample watched the same videos, with an additional video of an age-appropriate toy being activated by a human hand.

These videos were truncated (compared to the version used in Seattle) to a duration of 30–40 seconds. The number of trials (i.e., watched videos) depended on the infant’s cooperation. Both sites used a 128-channel Hydrocel geodesic sensor net and Electrical Geodesics (Eugene, Oregon) Net Station software. Scalp EEG was recorded at 500 Hz using a vertex reference and re-referenced offline using a robustly interpolated average. The London and the Seattle datasets were notch-filtered at 50 Hz and 60 Hz, respectively, to remove power line contamination.

Semi-automated pre-processing was done with the EEG-IP-Lossless pipeline (29) using an Octave interpreter running on a Compute Canada cluster. Pre-processing involved comprehensive data annotation to identify artifacts and non-stationarity in scalp channels and independent components. This pipeline provided an initial automated classification of the independent components as being either valid brain activity or capturing some artifacts, such as electromyographic, electrocardiographic, electrooculographic, and power line contamination. Quality control included an expert review of all data annotations and confirmation of artifacts informed by initial classification, topographies, activation time-series, dipole fit, and power spectrum. For an expanded description of pre-processing criteria and artifact thresholds, see (29,30). Once the flagged artifacts were removed from the data, the reconstituted scalp EEG was epoched into 1-second non-overlapping windows for source reconstruction and calculation of functional connectivity. We used relatively short time windows, as they have shown to be advantageous for estimating functional connectivity (34).

### Source reconstruction

Most EEG connectivity studies in autism have been performed on scalp electrode signals (17), which are known to have various limitations compared to source analyses, such as poorer signal-to-noise ratio, the impossibility to relate observations to brain structures, and the confounding effect of volume conduction, reference electrodes, and common sources (35–39).

Although tools for EEG source reconstruction are now widely available, they have been used only in a few autism studies (40,41). For infants with ASD or at high risk of ASD, the lack of age-matched templates has resulted in the use of head templates built from an adult population, such as the Montreal Neurological Institute (MNI) brain (41), which is likely to distort source estimations in ways that are not well-established. For this study, we used a recently developed set of infant structural head templates (42) to perform EEG cortical source reconstruction, investigate functional connectivity in infants, and identify potential ASD risk and resilience factors. To avoid confounding a potential effect of the head template with the recording time points, we used the 12-month template for all recordings. As a validation, the same analyses were performed with age-matched templates and resulted in qualitatively similar conclusions. The cortex for these templates has been parcellated using the Desikan-Killiany (43) scheme. Sources were estimated using MNE-Python 0.23 (44), with the eLORETA inverse operator (45), λ^2^=10^−4^, and with dipoles aligned perpendicular to the cortical mesh. Sources were averaged for every brain region, using the “mean flip” mode from MNE-Python.

### Functional connectivity

The corrected imaginary phase-locking value (CIPLV; Bruña et al., 2018) was computed between every pair of brain regions, as this measure is robust to the confounding impact of volume conduction and offers high test-retest reliability. Further, as functional connectivity estimates are biased depending on the sample size (Vinck et al., 2011; see also Supplementary Figure 1.a for the impact of sample size on CIPLV estimates), we ensured that estimates were all computed using the same number of epochs across subjects by bootstrapping the estimates using repeated samples of 20 epochs (see supplementary information for details). Recordings with less than 20 valid 1-s epochs have been rejected from the analyses.

We initially computed functional connectivity for the broadband signals (5–100 Hz), as well as for a few typical frequency bands: theta (5–8 Hz), alpha (8–12 Hz), beta (12–30 Hz), gamma (30–100 Hz). Given that our preliminary analyses did not indicate any noteworthy impact of frequency on between-group differences in connectivity, we report only the broadband analyses.

### Resting-state networks

To compare the functional connectivity estimated in this naturalistic video watching task within the different resting-state networks, we labeled the brain regions as being part of the auditory, default mode, dorsal attention, salience, or visual networks, or none of the above, following a previously published classification (48). The functional connectivity for each of these networks was computed as an average of the all-to-all connections between the regions that are part of the corresponding networks.

### Statistical analysis

Statistical analyses were run using pandas 1.1.4 for data manipulation, statsmodels 0.12.2 for linear regressions, and seaborn 0.11.0 and matplotlib 3.4.0 for visualization. To improve the normality of the connectivity measures, we transformed them using a logit equation (1).


(1) $$\text{logit}\left(\text{CIPLV}\right)=\text{log}\left(\frac{\text{CIPLV}}{1-\text{CIPLV}}\right)$$

This transformation changes the support of the connectivity measures from [0, 1] to [−inf, +inf] and helps diminish the asymmetry of the distribution, particularly the heavy right tail we observed in our empirical distributions. We further rejected the EEG recordings in which the functional connectivity was considered a statistical outlier, defined as being either more than 1.5 inter-quartile intervals above the third quartile or below the first quartile (see supplementary information for details). Table 2 lists the number of available recordings after artifact rejection.

We used mixed-effect multifactorial linear regressions to test the impact of biological sex, diagnostic groups, site, and age on functional connectivity, using the subject as grouping random-effect factor and the following model structure for the fixed effects:

(2) logit(CON)~sex*group+site*age

In a second time, we looked at correlations between ADOS calibrated severity scores and functional connectivity, within the ELA group using:

(3) logit(CON)~sex*ADOS+site*age

Regression (3) using ADOS includes only the ELA subjects, whereas the regression using the diagnostic group (2) used recordings from both groups.

## Results

Below, we proceed with a detailed investigation of the connectivity in the EEG-IP dataset, exploring categorical diagnostic group effects and the effect of ADOS scores as a dimension. We also investigated whether these relationships were affected by multiple factors, including age, biological sex, site, functional networks, and distance between communicating regions. Overall, as described in detail below, we observed a tendency for underconnectivity associated with familial risk and later ASD diagnosis, and we identified systematic differences between male and female ELA infants. These differences might reflect the higher resilience of females to ASD symptoms and support that a more pronounced alteration of the functional connectome is necessary for the symptomatic expression of ASD in females.

### Linear regressions

Both mixed-effect regression models described in (2,3) show a significant (2: p=2.4e-5; 3: p=1.4e-3) and negative effect of age on functional connectivity. The regressions using the diagnostic groups also revealed an interaction between the site and the age (p=0.03). All other factors were not significant (p>0.1). Model (2) was run with 350 observations from 179 participants, whereas model (3) was run with 131 observations from 71 participants (only including ELA infants with ADOS calibrated severity scores). In the following sections, we further explore these results to look for more specific effects (e.g., specific to age, network, etc.) that would not have been captured by these regression models.

### Age effect

Our data seem to show a decrease in overall connectivity with age, with potentially a U shape reaching a minimum value at 12 months. No other factor reliably modulated this effect.

### Regional specificity

**Figure 2.**
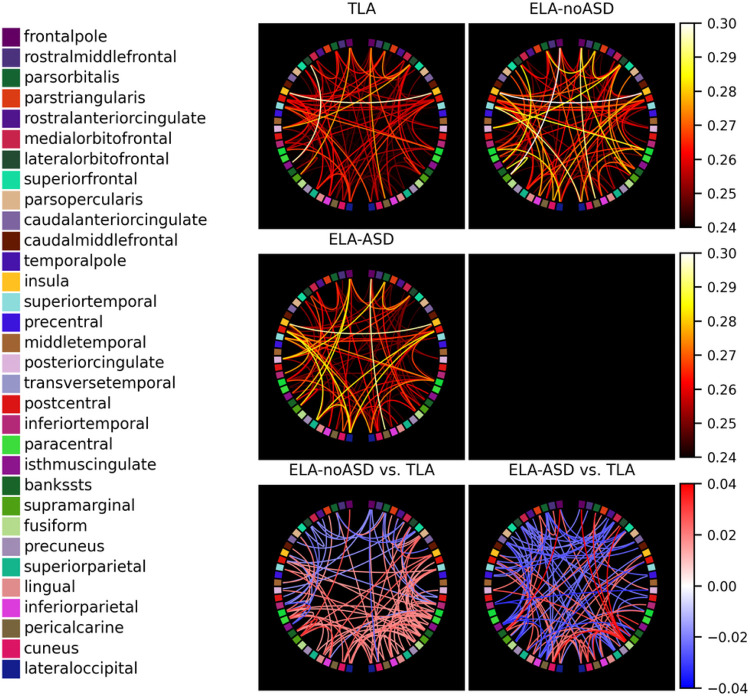
Functional connectivity (CIPLV) plots. Displayed for the three diagnostic groups (upper three graphs) and corresponding plots for the difference between the TLA and the two subgroups of ELA infants (lower two panels). The three top graphs and the two bottom graphs have been plotted using the same colormaps to allow fair comparisons. The left (right) side of these plots corresponds to the left (right) hemisphere. The order of the regions in each hemisphere is the same and is shown on the left side of the figure, from posterior (bottom) to anterior (top) regions. These plots only show the 100 region pairs with the strongest connectivity (top three panels) and the 100 pairs with the strongest between-group differences in connectivity (bottom two panels).

We further investigated brain region differences in functional connectivity. No clear topographic pattern of between-group differences emerging from this analysis (Figure 2).

**Figure 3.**
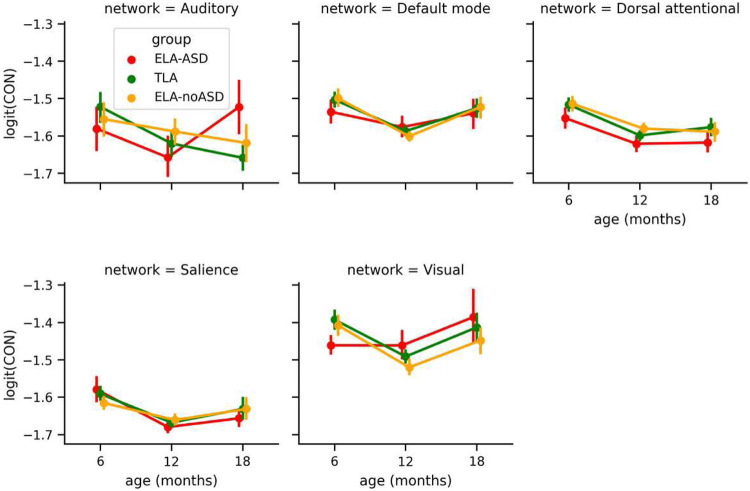
Logit-transformed CIPLV values within the different resting-state networks. Displayed per network (different panels), time points (x-axis), and diagnostic groups (color). Whiskers represent the bootstrapped 95% confidence interval.

Further, comparing average connectivity within resting-state networks did not support network-specific statistically significant group differences (Figure 3).

Similarly, we verified if between-group differences were modulated by the distance between brain regions, since there are reports of long-range underconnectivity and potentially short-range overconnectivity in ASD (17). Again, we did not see any evidence for such a relationship.

### Dimensional association with ADOS

**Figure 5.**
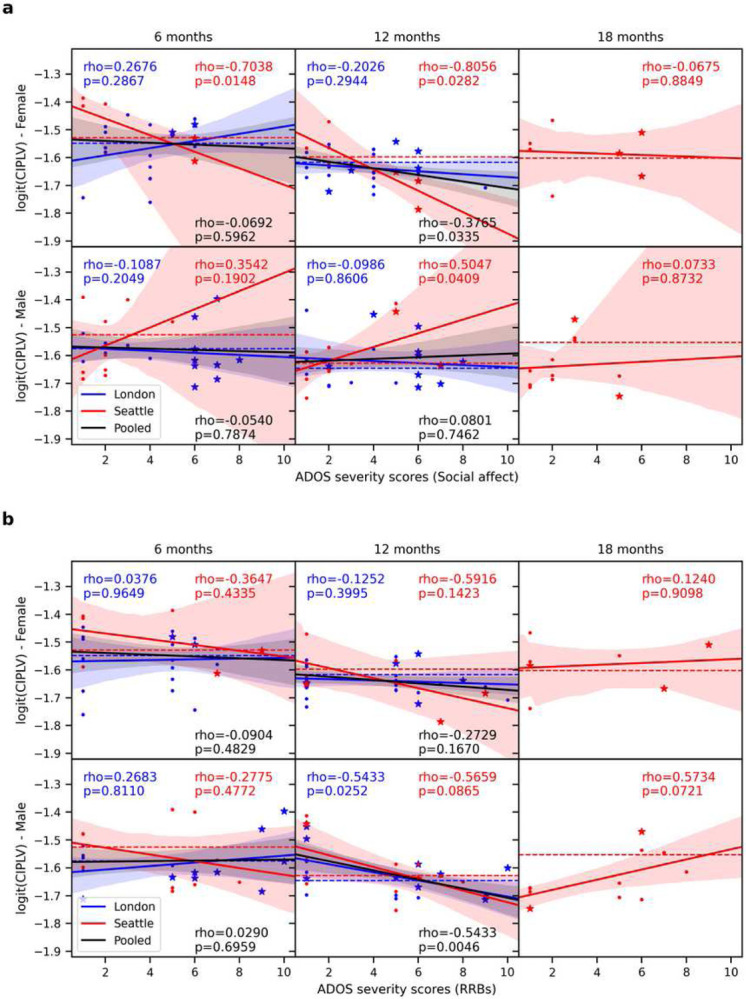
Regression between the logit-transformed CIPLV connectivity and ADOS calibrated severity scores for the ELA infants. Displayed per sex (rows), time point (columns), and sites (blue: London; red: Seattle; black: Pooled). The dashed lines indicate the average connectivity for TLA infants. Pearson’s coefficients of correlation (rho) are indicated, along with p-values (p) from robust linear regressions. Stars indicate participants diagnosed with ASD, whereas dots indicate neurotypical individuals. a) Social affect. b) RRBs. Overall ADOS severity scores are shown in supplementary figure 3.

Considering that our comprehensive analysis of group-level differences did not yield any striking difference, we examined dimensional associations within ELA between functional connectivity and ADOS calibrated severity scores (Figure 5). With pooled datasets, we observed a statistically significant negative correlation between functional connectivity at 12 months and social affect for females (but not males) (p=0.03; Pearson’s ρ=−0.38) and for restrictive and repetitive behaviors (RRBs) for males (but not females) (p=0.0046; Pearson’s ρ=−0.54).

It should be noted that many participants (52 in London; 36 in Seattle) have been assessed for ADOS at two time points, typically around 24 and 36 months. Since there are some differences in these scores at different time points (in particular, the London site shows low correlations between time points; see Supplementary Figure 4), differences in ADOS scores (e.g., due to lack of stability of ASD symptoms across the observation period leading particularly to false negative in early assessments (49)) could impact our results. The correlations we previously reported were based on the earliest ADOS scores available. To validate the impact of using ADOS at different time points, we also replicated our analysis using the ADOS scores from the oldest age. The two same observations were made, namely that functional connectivity was negatively correlated with social affect for females (but not males) (p=0.01; Pearson’s ρ=−0.50) and with RRBs for males (but not females) (p=0.01; Pearson’s ρ=−0.39). In this case, however, relationships were significant for the 6- instead of 12-month EEG (Supplementary Figure 5).

## Discussion

In this study, we used the EEG-IP database to examine whether functional connectivity between EEG cortical sources during the first year of life is atypical in ELA infants later diagnosed with ASD. It constitutes one of only a handful of studies on EEG functional connectivity in infants with ASD (24,25,27,28,50,51). Furthermore, it uses methods that improve upon past studies, such as using a robust functional connectivity metric and computing connectivity over cortical sources using age-matched head templates.

### Underconnectivity in infants with ASD

Our observations provide some insights into the developmental origins of underconnectivity in children and adults with ASD. They do not clearly support an underconnectivity hypothesis in very early childhood during the pre-diagnostic period, and correlations between ADOS and functional connectivity, when significant, tended to be of negative sign (i.e., larger ADOS were associated with less functional connectivity; see Figure 5 and Supplementary Figures 3 and 5). Nevertheless, our results are inconclusive with respect to a relationship between EEG functional connectivity and autism in infants. Any potential effect is likely to be of relatively small sizes or is difficult to capture through population averages due to sample heterogeneity. Such inconclusive results are compatible with recent reports from the large sample study LEAP (52).

Beyond generalized group differences in the all-to-all connectome, we also looked for different subsets of connections (e.g., belonging to specific resting-state networks) to investigate the possibility of a more specific neural effect. Our investigation failed to reveal a systematic, reliable, and reproducible pattern across our two data subsets. This situation may be due to a few factors. First, averages may not contrast groups if the effect of ASD on functional underconnectivity is inconsistent across subjects (e.g., an heterogeneous mixture of over and underconnectivity may end up to show a normal level of connectivity at the group level). Further, infant EEG data is inherently noisy, and the experimenters have little control over the infant’s behavior due to incontrollable factors such as tiredness and fussiness of the infants, which is likely to cause variable brain activations within and between subjects during EEG acquisitions. Lastly, we noted what looks like a significant degree of source leakage. Similar to volume conduction between scalp channels, source leakage generates zero-lag correlations between brain regions. It is hard to know what portion of unlagged synchrony is due to genuine zero-lag connectivity known to exist even between distant brain regions (53,54) and what part is due to source leakage and unresolved challenges associated with the under-determined estimation of cortical sources from scalp signals. Regardless of its cause, such source leakage blurs regional specificity by increasing the apparent similarity of brain activity across regions. Notwithstanding these potential issues and the possibility that we missed some effects, these analyses were thorough and extensive, and it is likely that any group effect, if present, would be of small size.

### Effect of biological sex on the relationship between ASD and functional connectivity

In our analyses, we observed different connectivity profiles between elevated-risk females and males, with females at 12 months showing a negative association between functional connectivity and social affect, as measured by ADOS calibrated severity scores. These conclusions are coherent with previous findings that females diagnosed with ASD show a larger deviation from neurotypical functional connectivity (55) and suggest that a larger difference compared to neurotypical functional connectivity is required for females to start showing ASD social symptoms. These differences could reflect early protective brain mechanisms that afford resiliency against ASD social symptoms in spite of altered connectivity in elevated-risk females, which may contribute to the lower prevalence of ASD in that population. The interplay of affected dimensions (RRBs versus social affect) and biological sex may also explain disparities in diagnosis prevalence.

Given the preponderance of autism in males, biological sex has emerged as a potential protective mechanism that mitigates risk (56). With respect to social affect symptoms, females appear to be more resilient, requiring higher genetic loading to reach ASD diagnostic threshold (57). Greater social cognitive abilities in females might contribute to such resilience and may be reflected in anatomical brain differences such as a comparatively thinner cortical sheet in several brain regions in females (58). A corresponding increased loading of functional connectivity required for ASD diagnosis was found in resting-state fMRI (55), with increased local connectivity in somatomotor and limbic networks and decreased local connectivity in default mode networks. Females with ASD have also been found to show increased connectivity compared to males (18) as well as typically developing females (19). Compared to males, higher functional connectivity in females has been observed for whole-brain connectivity and in functionally distinct networks, including the default mode and the central executive networks (15,16). In their study, Olson et al. (2020) found that reduced functional connectivity was associated with early ASD symptoms, specifically in females. Taken together, the functional connectivity profile in ASD suggests that higher connectivity in females is linked to lower severity of ASD symptoms, although there might be some regional specificity, at least in resting-state fMRI (55). Our findings in infants could reflect early neurodevelopmental markers of these divergent biological sex trajectories that perhaps contribute to reduced prevalence in females with higher functional connectivity. Such “markers” should be understood and studied for the insight that they can provide about underlying mechanisms rather than as potential clinically relevant biomarkers since the effect size of these relationships is likely to be relatively small, not making them plausible candidates for classification on their own^1^.

Lastly, we note that in this paper we follow the World Health Organization definitions for sex and gender, thus when discussing biological sex differences, we refer to differences currently thought to be influenced by biological and genetic properties. Still, we acknowledge that it is hard to completely disentangle the effects of biological sex and gender socialization in human development, particularly considering that gender socialization begins at birth and may influence neurobiology (59–61). We would also like to acknowledge that autistic individuals may be less likely to identify with their sex assignment from birth compared to neurotypical individuals (62,63), and while it is not possible to assess gender identity in infancy, we nonetheless encourage future autism studies to consider both gender and biological sex factors when possible.

## Limitations

This study has been limited mostly due issues related to small sample size inherent in sibling studies. In a previous systematic review, we have shown that studies on the impact of ASD on EEG and MEG functional connectivity often report contradictory results, probably due to many confounding factors across studies (e.g., differences in inclusion/exclusion criteria, in connectivity metrics, in frequency bands, in participant demographic characteristics) and methodological difficulties in estimating reliably the cerebral sources of EEG/MEG activity and the functional connectivity between them (17). This review also showed that small sample sizes are often used in studies of functional connectivity in autism. Histograms of sample sizes used in these studies show that samples of 10 ASD subjects or less are not uncommon (24%), and most studies (74%) have ASD groups of no more than 25 subjects (see Supplementary Figure 6).

Our study compares relatively well, with an average size for the ASD group of 22 participants^2^ across time points (sites combined). This is particularly true considering the prospective nature of this study, i.e., although a comparatively large number of infants enter the study at young ages, only a fraction of them are later diagnosed with ASD. For that reason, the non-autistic groups are significantly larger than the sample of participants with ASD. Actually, our dataset constitutes the largest infant sample and the second-largest sample overall among the functional connectivity studies included in our previous review.

Nevertheless, our analyses have been limited by relatively small sample sizes. The first reason for that is that infant EEG recordings are comparatively noisier than adult EEG recordings because it is harder to control sources of physiological artifacts (e.g., EMG and EOG contamination, movements, etc.) and to control the behavior or the attention of infants. Thus, a larger proportion of subjects end up discarded due to poor recording quality, and the data that are kept are generally more variable (noisy). The second reason is due to the unbalanced distribution of ASD versus comparison subjects that results from the prospective nature of studies in ELA infants. This imbalance causes much smaller effective sample sizes than the number of tested subjects (see the section *Effect of group imbalance on statistical power* in Supporting Material). Thus, in summary, although this study involved a large number of participants, our analyses are still limited by our sample size. This again stresses the need for future studies to reach larger effective sample sizes to support decorticating the complex interactions between the many factors (e.g., age, biological sex, diagnostic severity, brain region, frequencies) that may confound our understanding of the relationship between functional connectivity and autism. Further, improving the diversity of these samples (e.g., by including other early predictors of ASD such as a preterm birth or genetic conditions) may also be important to mitigate the possibility of ELA defined by sibling studies not being representative of other subgroups of autistic individuals.

## Conclusion

In summary, our analyses revealed relatively small and unspecific effects of ASD risk on EEG functional connectivity, potentially due to heterogeneity in how functional connectivity abnormalities present themselves in different subjects. It nevertheless showed sex-specific differences in how functional connectivity correlates with later autism severity scores. We obtained these results using a recently published connectivity measure (CIPLV) that solves many issues previously observed for similar measures. Further, we benefited from newly released structural head templates for infants to perform connectivity analysis between EEG sources rather than between EEG scalp signals, resulting in connectivity measures that can more easily be associated with brain regions and that are less likely to be confounded by known issues such as volume conduction and common sources. Our observations of ADOS scores negatively correlating with functional connectivity for social affect in females and RRBs in males might indicate that a different level of loading on functional connectivity is required to impact ADOS scores depending on the sex, which might highlight sex-specific resilience to social affect and RRBs symptoms in ASD.

## Figures and Tables

**Figure 1.**
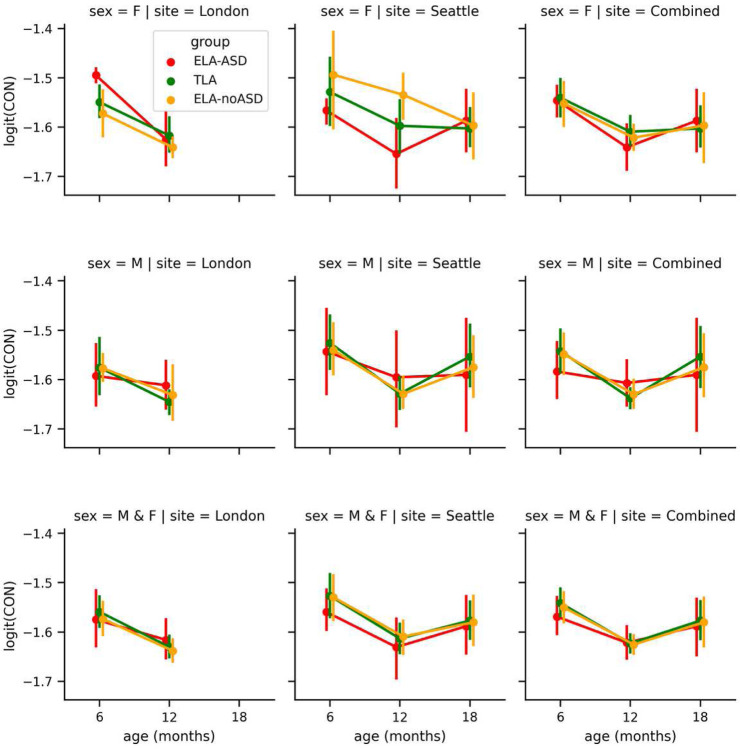
Average logit-transformed CIPLV connectivity. Displayed as a function of the age (x-axis), the site (columns), biological sex (row), and the diagnostic outcome groups (color). Whiskers represent the bootstrapped 95% confidence intervals. These plots are for all-to-all connectivity averaged by recording.

**Figure 4.**
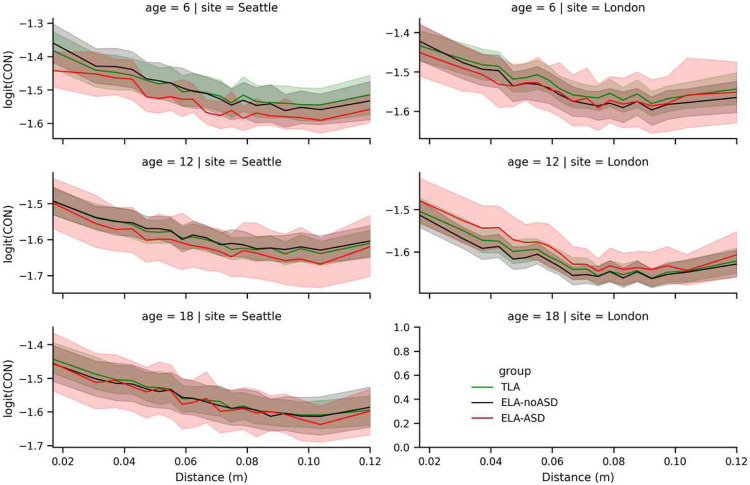
Average logit-transformed CIPLV connectivity as a function of the distance. Displayed between regions (x-axis), age (rows), site (columns), and group (color). To smooth these lines, distances are split into 20 bins each covering 5% of the distribution. Shaded regions show 95% bootstrapped confidence intervals.

**Table 1.** Sample sizes of the EEG-IP repository (male/female).

Risk Group	Outcome	London	Seattle	Total Across Sites
**ELA**	**ASD**	11/6	5/7	16/13
	**No ASD**	10/26	23/6	33/32
	**Unknown**	0/1	0/2	0/3
**ELA Total**		21/33	28/15	49/48
**TLA**	**ASD**	0/0	2/1	2/1
	**No ASD**	21/29	21/16	42/45
	**Unknown**	0/0	3/2	3/2
**TLA Total**		21/29	26/19	47/48
**Total across risk groups**		42/62	54/34	96/96

**Table 2.** Sample size available after artifact rejection, specified as male/female. Recordings were included if they had at least twenty 1s epochs of clean EEG and their estimated CIPLV was not a statistical outlier.

	6 months	12 months	18 months
**London**			
***TLA***	12/21	20/23	—
***ELA-noASD***	6/17	9/23	—
***ELA-ASD***	9/2	10/5	—
**Seattle**			
***TLA***	24/15	17/17	13/12
***ELA-noASD***	21/6	19/5	19/6
***ELA-ASD***	2/6	4/6	4/5
**Combined**			
***TLA***	36/36	37/40	13/12
***ELA-noASD***	27/23	28/28	19/6
***ELA-ASD***	11/8	14/11	4/5

## Data Availability

The datasets constituting EEG-IP and analyzed during the current study are available from the lead of the original studies, upon reasonable request to basis@bbk.ac.uk for the London dataset and to sjwebb@uw.edu for the Seattle dataset.

## Footnotes

1 Although these relationships do not have an effect size sufficiently strong to consider them as clinically relevant biomarkers, we cannot dismiss the possibility that they might eventually be useful in combination with other markers to better subtype ASD. However, much work would be needed before a robust classification from a constellation of weak markers significantly improves diagnostic practices currently based on simpler observational measures.

2 These averages are computed only for the two times points with data available at both sites (6 and 12 months).

## List of abbreviations

ADOS: Autism Diagnostic Observation Schedule
ASD: Autism Spectrum Disorder
CIPLV: Corrected Imaginary Phase-Locking Value
EEG: Electroencephalogram
EEG-IP: International Infant EEG Platform
ELA: Elevated likelihood for Autism
EOG: Electrooculogram
fMRI: functional Magnetic Resonance Imaging
MEG: Magnetoencephalogram
RRBs: Restrictive and Repetitive Behaviors
TLA: Typical likelihood for Autism
